# Is food insecurity related to health-care use, access and absenteeism?

**DOI:** 10.1017/S1368980019001885

**Published:** 2019-08-06

**Authors:** António Melo, Maria Ana Matias, Sara S Dias, Maria João Gregório, Ana M Rodrigues, Rute Dinis de Sousa, Helena Canhão, Julian Perelman

**Affiliations:** 1 Escola Nacional de Saúde Pública, Universidade NOVA de Lisboa, Avenida Padre Cruz, 1600-560 Lisbon, Portugal; 2 Nova School of Business and Economics, Universidade NOVA de Lisboa, Lisbon, Portugal; 3 EpiDoC Unit, Centro de Estudos de Doenças Crónicas (CEDOC), NOVA Medical School, Universidade NOVA de Lisboa (NMS/UNL), Lisbon, Portugal; 4 EpiSaúde Scientific Association, Évora, Portugal; 5 Center for Innovative Care and Health Technology, ciTechCare, School of Health Sciences, Polytechnic Institute of Leiria, Leiria, Portugal; 6 Faculdade de Ciências da Nutrição e Alimentação, Universidade do Porto, Porto, Portugal; 7 Programa Nacional para a Promoção da Alimentação Saudável, Direção-Geral da Saúde, Lisbon, Portugal; 8 Sociedade Portuguesa de Reumatologia, Lisbon, Portugal; 9 Rheumatology Research Unit, Instituto de Medicina Molecular, Lisbon, Portugal; 10 Serviço Reumatologia, Centro Hospitalar Lisboa Central, Lisbon, Portugal; 11 Centro de Investigação em Saúde Pública, Escola Nacional de Saúde Pública, Universidade NOVA de Lisboa, Lisbon, Portugal

**Keywords:** Food insecurity, Health-care use, Health-care access, Absenteeism

## Abstract

**Objective:**

Food insecurity (FI) is defined as uncertain access to healthy food in quantity and quality. We hypothesize that FI may be associated with greater health-care use and absenteeism because it may amplify the effect of diseases; also, FI may be associated with reduced health-care access because it reflects economic vulnerability. The present study estimates the association between FI and health-care use and access, and absenteeism.

**Design:**

Cross-sectional data collected in 2015–2016. Health-care use was measured as the number of consultations, taking any drug and having been hospitalized in the past year. Health-care access was measured by the suspension of medication and having fewer consultations due to financial constraints. Absenteeism was measured by the weeks of sickness leave. Binary variables were modelled as a function of FI using logistic regressions; continuous variables were modelled as a function of FI using negative binomial and zero-inflated negative binomial regressions. Covariates were included sequentially.

**Setting:**

Portugal.

**Participants:**

Non-institutionalized adults from the EpiDoc3 cohort (*n* 5648).

**Results:**

FI was significantly associated with health-care use before controlling for socio-economic conditions and quality of life. Moderate/severe FI was positively related to the suspension of medicines (adjusted OR = 4·68; 95 % CI 3·11, 6·82) and to having fewer consultations (adjusted OR = 3·98; 95 % CI 2·42, 6·37). FI and absenteeism were not significantly associated.

**Conclusions:**

Our results support the hypothesis that FI reflects precariousness, which hinders access to health care. The greater use of health care among food-insecure people is explained by their worse quality of life and lower socio-economic condition, so that the specific role of poor nutrition is unclear.

According to the FAO, food insecurity (FI) is defined by ‘a situation that exists when people lack secure access to sufficient amounts of safe and nutritious food for normal growth and development and an active and healthy life’ (p. 50)^(^ ^1^^)^. This definition includes multiple dimensions (food availability, food access, food utilization and stability) which are complex to evaluate using simple quantitative questions on a larger scale, so that common tools used to measure FI are focused on food access related to the economic dimension^(^ ^2^^,^ ^3^^)^.

FI, according to its economic-related aspects, is related to different nutritional outcomes, either undernutrition or overnutrition. Lower nutrient intakes are shown to be common among adults with FI. However, studies have also shown that FI may coexist with obesity. Bhattacharya *et al.*^(^ ^4^^)^ showed that food-insecure people had less healthy diets, were more prone to have lower serum nutrient levels and were more likely to be obese. Obesity among food-insecure people might be explained by the increased consumption of affordable energy-dense food^(^ ^4^^,^ ^5^^)^, thus showing that FI is not merely a condition of insufficient food quantity but also of incapacity to have a healthy diet. Notable is that the association between FI and obesity is gender- and age-dependent, with a stronger association among women^(^ ^6^^)^. Given that poor dietary intake is related to poor health, poor immunity and development of chronic diseases^(^ ^7^^,^ ^8^^)^, it is expected that people who suffer from FI have worse health outcomes.

Thus, FI, given its link with poor health, might lead to negative externalities like increased health-care utilization and absenteeism, which increase the perceived burden of FI on the economy. For example, in Ontario, Canada, Tarasuk *et al.*^(^ ^9^^)^ observed that households’ FI was associated with higher health-care utilization and costs.

However, a reverse mechanism may also occur. Having poor health may predispose people to become food insecure or aggravate their FI^(^ ^10^^)^, since the need for medical care possibly competes with the need for food, a condition usually referred to as ‘treat or eat’^(^ ^11^^)^. This bidirectional link between FI and health might culminate in hindered access to health care. Existing evidence shows, for example, that food-insecure households are more likely to suffer from medication underuse^(^ ^11^^,^ ^12^^)^. Specifically, Berkowitz *et al.*^(^ ^11^^)^ estimated that one-third of chronic disease patients in the USA struggled to buy food, medication or both, which corroborates the hypothesis that FI can be associated with decreased access to health care.

Kushel *et al.*^(^ ^13^^)^ provided insights on the relationship between FI and both health-care access and use among low-income American adults. Using a national representative sample, they found that being food insecure was positively related to inpatient stays and emergency department visits. In addition, the authors found a positive relationship between FI and having suspended medication and postponing needed medical care, a sign that FI is also associated with poor access to health care in the USA.

Regarding the relationship between FI and work productivity, there are, to the best of our knowledge, no studies on the subject, although the literature has been putting forward the hypothesis of a negative association^(^ ^14^^,^ ^15^^)^. The reasoning is that, by increasing the likelihood of being sick, FI can decrease the number of working days due to increases in the number and duration of sickness leaves. Besides, it can decrease productivity since workers have to conduct their work under suboptimal physical conditions. Reversely, Heflin *et al.*^(^ ^16^^)^ put forward the hypothesis that more working hours per week is associated with higher income or other unobservable characteristics such as family structure or tenacity, which decrease the likelihood of suffering from FI. There is some work on how having a healthy diet is associated with reduced absenteeism (missing work due to sickness)^(^ ^17^^)^ and how interventions to improve workers’ diet within companies translate into decreased absenteeism and presenteeism (work underperformance due to sickness)^(^ ^18^^)^. Both findings suggest an association between FI and productivity.

Therefore, we developed two hypotheses. First, FI may be associated with greater health-care use and absenteeism because it may amplify the effect of diseases, thus not having a relationship with health-care access. Second, FI may be associated with reduced access to health care because it reflects economic vulnerability, which may also translate into reduced access to health care. Certainly, this second hypothesis depends on health systems; we expect lower access to care being more likely in countries where it is related to the ability to pay than in countries with universal free health care. The case of Portugal is not straightforward, as the universal National Health Service (NHS) suffers from important weaknesses, so that many people use the private system, creating severe inequities in health-care use^(^ ^19^^)^.

In Portugal, our work group^(^ ^3^^)^ estimated that approximately one in five persons suffers from FI. In the present study we examine the association between FI and health-care use and access, and between FI and absenteeism, using data generated by the nationally representative Epidemiology of Chronic Diseases Cohort Study (EpiDoC3) survey, collected between 2015 and 2016 in Portugal with 5648 non-institutionalized adults.

## Methods

### Data

We used data of the EpiDoC3, the third wave of data collection of a nationally representative sample of 5648 non-institutionalized adults collected between September of 2015 and July of 2016, with the purpose of studying the determinants and outcomes of health. Rodrigues *et al.*^(^ ^20^^)^ and Gregório *et al.*^(^ ^3^^)^ provide additional information about the EpiDoC cohort, specifying how the data were collected, how each variable was measured, the ethics committee approval and detailed descriptive statistics of each variable.

### Dependent variables

We modelled health-care utilization using the self-reported number of hospitalizations, medical appointments and taking medication. Health-care access was measured using the suspension of medication and having to cut consultations due to financial constraints. Specifically, two ‘yes’ or ‘no’ questions were asked: ‘In the last 12 months, did you reduce the number of medical appointments or stop attending appointments because it was too expensive?’ and ‘In the last 12 months, did you skip or stop taking medication because it was too expensive?’. Finally, absenteeism was measured by number of weeks of sickness leave.

### Explanatory variables

Our variable of interest, FI, was measured through a psychometric household food insecurity scale validated for the Portuguese population^(^ ^21^^)^ that was adapted from the Brazilian FI survey and based on the previous work conducted by Radimer *et al.*^(^ ^22^^,^ ^23^^)^, Campbell^(^ ^24^^)^ and the Community Childhood Hunger Identification Project^(^ ^25^^)^. This scale focused on the 3 months prior to the FI survey and is composed of eight questions for households without children and fourteen for households with children. As an example, there were the two following questions: ‘Have you worried that your household runs out of food before you have enough money to buy more?’ and ‘Has any household member skipped a meal for not having enough money?’^(^ ^3^^)^. This FI survey enables the classification of respondents into one of four degrees of food security: (i) food secure, (ii) mildly food insecure, (iii) moderately food insecure and (iv) severely food insecure. Since there were few observations of both moderate and severe FI categories (286 and 144, respectively), we decided to merge them. For a more detailed description on the construction of this measure see Gregório *et al.*^(^ ^3^^)^.

### Covariates

We used as covariates aspects related to the individual’s demographic and socio-economic profile, namely age (treated as continuous in order to capture the average effect on health-care use and access) and gender. These variables were included to avoid a possible confounding bias, as we expect older people and women to be more frequent health-care users and to suffer more from FI. We also included economic insecurity (people were asked to rate their economic condition as ‘strongly secure’, ‘secure’, ‘insecure’ and ‘strongly insecure’, which we coded as 0 if the person was economically secure and 1 otherwise); educational level (more than 12 years of education; between 10 and 12 years; between 5 and 9 years; less than 5 years); employment status (employed, unemployed, retired, student, homemaker, suffering from temporary incapacity to work); and whether they had private insurance (people were asked to detail their health insurance system, which we coded as 0 for those who benefited only from the NHS and 1 for those covered by any other health insurance system). These variables were considered as potential confounders because it is very likely that people suffering from food insecurity have a low socio-economic status, while a low socio-economic status is known to increase health-care use^(^ ^26^^)^.

The survey also integrated the Portuguese validated version of the European Quality of Life – 5 Dimensions questionnaire (EQ-5D)^(^ ^27^^,^ ^28^^)^, which measures health-related quality of life on a scale from 0 to 1, where 0 is death and 1 is the best possible health state, although negative values are also accepted for states worse than death.

### Statistical methods

We modelled access, use and productivity for each person *i* as a function of FI and other covariates:

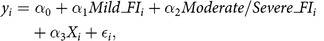

(1)
where *α*_0_ is the constant term; *α*_1_ to *α*_3_ are the regressors’ coefficients; *ϵ*_i_ is the error term; *Mild_FI*_*i*_ is a dummy variable that takes the value 1 if person *i* is mildly food insecure, and 0 otherwise; *Moderate/Severe_FI*_*i*_ is a dummy variable that takes the value 1 if person *i* is moderately or severely food insecure, and 0 otherwise; and *X*_*i*_ is a vector of covariates (age; age squared; female; educational level; professional status; economic insecurity; health-related quality of life; having insurance). All analyses were performed by introducing the independent variables sequentially.

For the binary dependent variables (being hospitalized, taking medication, suspending medication, reducing the number of consultations), we applied logistic regressions. Regarding the number of appointments and given the overdispersed distribution (variance higher than the mean) and excess of zeros (10 % of the sample was zeros), we used a negative binomial regression^(^ ^29^^)^.

Following Fitzgerald *et al.*^(^ ^17^^)^ in their study of the association between productivity and diet, for the number of weeks of sickness leave we used a zero-inflated negative binomial regression since the variable is overdispersed and has a great number of zeros (79·6 %). The zero-inflated negative binomial regression suits our data well because it takes into account two potential mechanisms that generate zeros: (i) being sick and not using sickness leave; and (ii) being healthy and not needing sickness leave. In this two-part model, we first estimated the likelihood of a person being healthy and not being absent (the inflated model) by applying a logistic regression. Then, we used a negative binomial regression to estimate the likelihood of the number of weeks of sickness leave that a person took, as depicted in equation (1)^(^ ^29^^,^ ^30^^)^. We confirmed that both variables were overdispersed by applying the test for overdispersion^(^ ^31^^)^.

## Results

### Descriptive statistics

People with severe FI were on average older, with a worse quality of life, and went to the doctor more often. We did not find any statistically significant difference across FI levels regarding missing work days (Table 1).

**Table 1:** Participant characteristics by food insecurity category (continuous variables) among non-institutionalized adults from the EpiDoc3 cohort (*n* 5648), September 2015–July 2016†

	Sample observations (*N*)	Overall		Food insecurity			
		Mean	sd	Secure (mean)	Mild (mean)	Moderate/severe (mean)	*F* test (equal means)
Age (years)	5648	49·64	18·11	48·36	52·49	58·15	36·81***
Health-related quality of life (EQ-5D)	5648	0·78	0·29	0·83	0·70	0·48	129·36***
No. of appointments	4949	4·39	5·18	4·13	5·76	6·08	19·49***
No. of weeks of sickness leave	2417	0·88	3·86	0·79	1·39	1·67	2·45*

EpiDoC3, Epidemiology of Chronic Diseases Cohort Study; EQ-5D, European Quality of Life – 5 Dimensions questionnaire.

* *P <* 0·10, ***P < 0·01.

† The presented means and sd are estimates for the population. The null hypothesis of the *F* test is that all food insecurity categories’ means are equal, per variable.

Food-insecure people represented 19 % of the sample (Table 2). Among economically insecure people, 44·8 % suffered from FI whereas the prevalence of FI among economically secure people was 6·6 %. Both mild and moderate/severe FI had the highest prevalence among people who only completed primary school (30·1 %), contrasting with people who attended college (6·0 %). FI was also more frequent among unemployed people (32·3 %), retired people (24·5 %), women (23·5 %) and people without health insurance (23·6 %). In addition, 27·8 and 23·5 % of people who had been hospitalized or took medication, respectively, suffered from FI. People who for economic reasons suspended medication or decreased the number of medical appointments had an FI prevalence of 59 and 51 %, respectively, compared with 41 and 49 % among people without FI. All proportions proved to be statistically significantly different from each other across the different categorical variables.

**Table 2:** Prevalence of food insecurity by participant characteristics and food insecurity category among non-institutionalized adults from the EpiDoc3 cohort (*n* 5648), September 2015–July 2016

		Food insecurity						
		Secure		Mild		Moderate/severe		*χ*^2^
		*n*†	%‡	*n*†	%‡	*n*†	%‡	
Sex	Male	1645	85·3	247	10·6	100	4·1	69·31***
	Female	2506	76·5	703	17·2	330	6·3	
Education	College	966	94·0	57	4·8	15	1·2	347·14***
	High school	862	84·6	147	13·8	27	1·6	
	Middle school	795	76·7	226	16·5	81	6·9	
	Primary school or less	1513	69·9	512	20·0	297	10·1	
Professional status	Employed	2004	87·0	337	10·7	77	2·3	209·29***
	Retired	1434	75·5	337	16·3	186	8·2	
	Unemployed	299	67·7	127	23·5	74	8·8	
	Others§	408	74·6	148	15·8	92	9·6	
Economic insecurity	Secure	3003	93·4	257	6·0	25	0·6	1146·25***
	Insecure	1148	56·2	693	29·6	405	14·2	
Insurance	Uninsured	2570	76·4	744	16·5	374	7·1	138·89***
	Insured	1559	88·9	200	9·5	53	1·6	
Hospitalized	No	3652	81·7	800	13·4	357	4·9	33·49***
	Yes	498	72·1	150	19·4	73	8·4	
Took medication	Did not take	1458	85·8	255	11·4	72	2·8	90·08***
	Took	2690	76·5	693	16·3	358	7·3	
Suspended medication	Did not suspend	3974	83·3	782	12·9	269	3·8	477·90***
	Suspended	168	40·7	165	32·4	159	26·9	
Reduction of appointments	Did not reduce	3924	82·9	795	13·0	298	4·2	293·35***
	Reduced	223	48·9	154	30·3	131	20·8	
Total		4151	80·7	950	14·1	430	5·3	

EpiDoC3, Epidemiology of Chronic Diseases Cohort Study.

*** *P <* 0*·*01.

† Absolute frequencies.

‡ Weighted percentages per category.

§ Includes students, household work and people with temporary incapacity.

### Multivariate analysis

The number of consultations and having been hospitalized were strongly associated with FI (Models 1 and 4 of Table 3), but when adjusting for health-related quality of life and socio-economic characteristics (Models 2, 3, 5 and 6), associations with mild or moderate/severe FI became non-significant (Table 3). In addition, people who suffered from economic insecurity displayed a statistically significant and positive association with all measures of health-care utilization.

**Table 3:** Association between food insecurity and health-care use among non-institutionalized adults from the EpiDoc3 cohort (*n* 5648), September 2015–July 2016†,‡

	Hospitalization (yes/no)			No. of appointments			Took medication (yes/no)		
	Model 1	Model 2	Model 3	Model 4	Model 5	Model 6	Model 7	Model 8	Model 9
	OR	AOR	AOR	*β*	*β*	*β*	OR	AOR	AOR
Mild	1·523***	1·332*	1·176	0·241***	0·125*	0·049	1·091	0·989	0·887
95 % CI	1·130, 2·053	0·993, 1·788	0·852, 1·623	0·109, 0·372	–0·008, 0·258	–0·090, 0·178	0·805, 1·479	0·721, 1·356	0·628, 1·252
Moderate or severe	1·730***	1·180	1·000	0·231***	0·014	–0·081	1·210	0·885	0·764
95 % CI	1·177, 2·544	0·786, 1·770	0·649, 1·539	0·082, 0·379	–0·136, 0·165	–0·239, 0·076	0·820, 1·787	0·585, 1·340	0·491, 1·189
Health-related quality of life (EQ-5D)		0·235***	0·252***		–0·797***	–0·767***		0·285***	0·307***
95 % CI		0·157, 0·351	0·167, 0·381		–0·985, –0·610	–0·954, –0·581		0·173, 0·471	0·185, 0·509
Economic insecurity			1·433^∗∗^			0·200***			1·347**
95 % CI			1·078, 1·906			0·076, 0·324			1·054, 1·722
*N*	5458	5458	5428	4788	4788	4761	5454	5454	5424
Type of regression	Logit	Logit	Logit	NB	NB	NB	Logit	Logit	Logit

EpiDoC3, Epidemiology of Chronic Diseases Cohort Study; AOR, adjusted odds ratio; EQ-5D, European Quality of Life – 5 Dimensions questionnaire; NB, negative binomial.

* *P <* 0*·*10, **P < 0*·*05, ***P < 0*·*01.

† All regressions are adjusted for age, sex, education level, professional status and having some type of health insurance.

‡ Models 1, 4 and 7: health-care use is modelled as a function of food insecurity; Models 2, 5 and 8: health-care use is modelled as a function of food insecurity, adjusting for quality of life; Models 3, 6 and 9: health-care use is modelled as a function of food insecurity, adjusting for quality of life and economic insecurity.

Suffering from mild FI (adjusted OR (AOR) = 2·46; 95 % CI 1·79, 3·40) and moderate/severe FI (AOR = 4·68; 95 % CI 3·11, 6·82) increased the likelihood of suspending medication. We found a strong association of mild FI (AOR = 2·39; 95 % CI 1·68, 3·40) and moderate/severe FI (AOR = 3·98; 95 % CI 2·49, 6·37) with reduced number of medical appointments due to financial constraints. The magnitude of the associations was higher for the most severe cases of FI. Economically insecure people were also more likely to suspend medication (AOR = 2·87; 95 % CI 2·04, 4·04) and to reduce the number of appointments (AOR = 2·25; 95 % CI 1·56, 3·26). Besides, adjusting for socio-economic variables and quality of life reduced the magnitude of the coefficients but not their statistical significance (Models 3 and 6 of Table 4).

**Table 4:** Association between food insecurity and health-care access among non-institutionalized adults from the EpiDoc3 cohort (*n* 5648), September 2015–July 2016†,‡

	Suspended medication			Reduced medical appointments		
	Model 1	Model 2	Model 3	Model 4	Model 5	Model 6
	OR	AOR	AOR	OR	AOR	AOR
Mild	3·824***	3·529***	2·463***	3·451***	3·217***	2·389***
95 % CI	2·810, 5·204	2·608, 4·775	1·785, 3·398	2·490, 4·785	2·336, 4·429	1·677, 3·402
Moderate or severe	8·979***	7·103***	4·680***	6·909****	5·651***	3·984***
95 % CI	6·241, 12·916	4·842, 10·418	3·112, 6·824	4·560, 10·467	3·697, 8·638	2·491, 6·372
Health-related quality of life (EQ-5D)		0·378***	0·427***		0·432****	0·491***
95 % CI		0·248, 0·576	0·279, 0·653		0·287, 0·651	0·322, 0·748
Economic insecurity			2·871^∗∗∗^			2·254***
95 % CI			2·039, 4·042			1·557, 3·263
*N*	5445	5445	5416	5453	5453	5423
Type of regression	Logit	Logit	Logit	Logit	Logit	Logit

EpiDoC3, Epidemiology of Chronic Diseases Cohort Study; AOR, adjusted odds ratio; EQ-5D, European Quality of Life – 5 Dimensions questionnaire.

*** *P <* 0*·*01.

† All regressions are adjusted for age, sex, education level, professional status and having some type of health insurance.

‡ Models 1 and 4: health-care access is modelled as a function of food insecurity; Models 2 and 5: health-care access is modelled as a function of food insecurity, adjusting for quality of life; Models 3 and 6: health-care access is modelled as a function of food insecurity, adjusting for quality of life and economic insecurity.

Suffering from FI or being economically insecure had no association with the number of weeks of sickness leave (Table 5). Healthiness levels displayed a negative association with absenteeism; hence, people who had better health were less prone to miss work due to sickness.

**Table 5:** Association between food insecurity and absenteeism among non-institutionalized adults from the EpiDoc3 cohort (*n* 5648), September 2015–July 2016†,‡

	No. of weeks of sickness leave		
	Model 1	Model 2	Model 3
	OR	AOR	AOR
Mild	1·190	0·895	0·829
95 % CI	0·698, 2·029	0·530, 1·512	0·486, 1·411
Moderate or severe	1·156	0·947	0·883
95 % CI	0·615, 2·171	0·503, 1·781	0·465, 1·676
Health-related quality of life (EQ-5D)		0·298***	0·303***
95 % CI		0·126, 0·704	0·126, 0·726
Economic insecurity			1·154
95 % CI			0·763, 1·747
*N*	2373	2373	2363
Type of regression	ZINB	ZINB	ZINB

EpiDoC3, Epidemiology of Chronic Diseases Cohort Study; AOR, adjusted odds ratio; EQ-5D, European Quality of Life – 5 Dimensions questionnaire; ZINB, zero-inflated negative binomial.

*** *P <* 0·01.

† All regressions are adjusted for age, sex, education level, professional status and having some type of health insurance.

‡ Model 1: absenteeism is modelled as a function of food insecurity; Model 2: absenteeism is modelled as a function of food insecurity, adjusting for quality of life; Model 3: absenteeism is modelled as a function of food insecurity, adjusting for quality of life and economic insecurity.

## Discussion

We found FI to be associated with hindered access to health care, in accordance with previous research^(^^11^^,^^13^^)^. By contrast, we found no evidence of an independent association between FI and health-care use, or between FI and absenteeism. Note, however, that FI was strongly associated with the number of consultations and being hospitalized until we adjusted for quality of life, which highly reduced the association, and for socio-economic covariates, which eliminated it.

The non-significant association with health-care use contradicted the findings obtained by Kushel *et al.*^(^^13^^)^ and Tarasuk *et al.*^(^^9^^)^. First, we employed a more detailed measure of FI, which makes our results hardly comparable to those obtained by Kushel *et al.*^(^^13^^)^. As for Tarasuk *et al.*^(^^9^^)^, the authors measured utilization as a dichotomous variable while we employed three measures of utilization. Specifically, our measures not only allow differentiating the type of care but also taking into account the intensity of utilization^(^^32^^)^. Also, Tarasuk *et al.*^(^^9^^)^ did not control for the individuals’ health conditions, as we did. Moreover, to measure health-care use, Tarasuk *et al.*^(^^9^^)^ used administrative health data on food-insecure people, while we had self-reported assessments of utilization, which might contribute for the different results. Hence, the different nature of our measures makes comparisons hard to establish.

Note that even though poor nutrition and having a low socio-economic status might lead to poor health^(^^7^^,^^8^^,^^33^^,^^34^^)^, which may be related to high levels of health-care utilization and absenteeism, this association was not reflected through FI in our results.

Let us recall our two hypotheses. First, a positive relationship between FI and health-care use and absenteeism was expected due to poor health. Second, a hindered access was expected because FI is associated with economic vulnerability and the ‘treat or eat’ dilemma. Our results partially support the first hypothesis and clearly support the second hypothesis.

First, the magnitude of the association between FI and health-care use was reduced when we introduced quality of life in the regression model. On the one hand, this result may signify that FI provokes a worse health condition (i.e. quality of life), which leads people to seek for more care. This interpretation would be in line with our first hypothesis. On the other hand, the association reduced even more and lost significance when economic insecurity was introduced in the regression model. Hence, it may be that both FI and higher care are a consequence of a worse socio-economic condition, so that there would be no real causal pathway from FI to health-care use. In fact, the literature points to a strong association between FI status and individuals’ socio-economic characteristics, specifically income^(^^35^^,^^36^^)^, education, household size, house ownership^(^^36^^)^, unemployment status and childhood economic insecurity. Thus, FI serves as a direct measurement of well-being^(^^36^^)^ that may contribute to depict a more precise picture of a person’s socio-economic profile, given that not only poor households suffer from FI^(^^36^^)^. Hence, assessing FI levels might be important to understand material deprivation across different layers of society, contributing to the well-known evidence on socio-economic inequalities in health and health care.

Second, the lack of access suggests the occurrence of the ‘treat or eat’ phenomenon, a sign of enhanced socio-economic vulnerability. Although we thought that the association between FI and access would be mitigated by the existence of a universal NHS, even in a universal health-care system there are costs that might discourage people with low socio-economic status to seek care. In Portugal, Barros *et al.*^(^^37^^)^ found that medication and co-payments were the highest costs for the patient when seeking health care. Indeed, if co-payments are low and many people are exempt for consultations, this is not the case for drugs, with high co-payments and no exemptions. If these costs are competing with food, then FI constitutes a barrier to access.

Regarding productivity, theoretical considerations suggest a positive association between FI and absenteeism, a result that we did not confirm. A possible explanation is the simultaneous occurrence of the two hypotheses. On the one hand, food-insecure people may be more likely to miss work for being more prone to be sick; on the other hand, they may attend work in suboptimal health conditions in order to ensure economic security. In Portugal, individuals lose some of their income if they miss days at work even if they present a medical justification (Artigo 255º do Código do Trabalho). These two events may have an opposite effect on absenteeism.

Hence, we cannot conclude that FI generates negative externalities on the economy either through a higher consumption of health resources or through decreases in productivity due to absenteeism. Still, it is associated with hindered access to health care, highlighting that universal coverage through the NHS might not be sufficient to overcome inequalities in health care.

The present study has some limitations. First, our results suffer from recall bias since we are using self-reported data. People recall absenteeism or levels of health-care utilization more accurately if the recall period is short^(^^38^^)^. As we measure absenteeism in weeks, we should not suffer from a major recall bias in our productivity analysis. Regarding the number of appointments, our results may be more subject to recall bias, given that we used the annual number of appointments. However, annual experiences such as hospitalizations were shown to be recalled precisely^(^^38^^)^, and no bias is expected in what concerns the self-reporting of taking medication, as the question asks whether a person was currently undergoing any medication. Second, we worked with a cross-sectional data set which does not allow to infer a causal relationship between FI, health-care access, use and productivity. Third, the survey used to measure FI includes questions that are linked to economic insecurity, so that it is difficult to disentangle the nutrition component of FI from its socio-economic component. Nevertheless, our understanding is that the concept of FI is beyond lack of nutritional intake but instead, a multidimensional indicator that reflects various levels of precariousness. Fourth, the questions related to FI and the outcome variables have a different time span. While FI was asked relative to the last 3 months prior to the survey, the outcome variables recalled the last 12 months. Therefore, we assumed that the individuals’ FI status remained constant throughout the year, which is very plausible but may not be always the case.

According to our results, food-insecure households in Portugal face barriers in access that can be addressed by implementing policies for which there is evidence of FI mitigation, namely food assistance programmes that are nutritionally adequate^(^^36^^,^^39^^)^ and other safety net programmes for vulnerable groups like the unemployed^(^^40^^)^, the elderly^(^^41^^)^ and low-income families^(^^42^^)^. That is, those groups who had the highest prevalence of FI in our study. Food-insecure people might also benefit from workplace nutritional interventions for which there is strong evidence of improved workers’ diet and productivity^(^^17^^,^^18^^)^. These policies have the potential of decreasing FI and increasing access to health care. Their implementation may no longer force people to have to choose between food and health care.

Overall, and in line with previous studies, we found a positive and strong association between FI and hindered access to health care, which suggests that FI might be a proxy of precariousness, thus reinforcing its multidimensional relevance beyond its role as a nutritional measure. These results highlight that the ‘treat or eat’ dilemma may be a reality despite the universal health-care coverage through an NHS. The greater use of health care among food-insecure people is fully explained by their worse quality of life and lower socio-economic condition, so that the specific role of poor nutrition is unclear.
